# An ISFET Microarray Sensor System for Detecting the DNA Base Pairing

**DOI:** 10.3390/mi12070731

**Published:** 2021-06-22

**Authors:** Peng Sun, Yongxin Cong, Ming Xu, Huaqing Si, Dan Zhao, Dongping Wu

**Affiliations:** 1 State Key Laboratory of ASIC and System, School of Microelectronics, Fudan University, Shanghai 200433, China; 18112020068@fudan.edu.cn (P.S.); 15210720050@fudan.edu.cn (Y.C.); xuming123@263.net (M.X.); 17112020035@fudan.edu.cn (H.S.); 2 Shanghai Turtle Technology Company Limited, Shanghai 200439, China; zhaodan@fudan.edu.cn

**Keywords:** DNA base pairing, ISFET microarray chip, high resolution, pH measurement, microfluidic system

## Abstract

Deoxyribonucleic acid (DNA) sequencing technology provides important data for the disclosure of genetic information and plays an important role in gene diagnosis and gene therapy. Conventional sequencing devices are expensive and require large and bulky optical structures and additional fluorescent labeling steps. Sequencing equipment based on a semiconductor chip has the advantages of fast sequencing speed, low cost and small size. The detection of DNA base pairing is the most important step in gene sequencing. In this study, a large-scale ion-sensitive field-effect transistor (ISFET) array chip with more than 13 million sensitive units is successfully designed for detecting the DNA base pairing. DNA base pairing is successfully detected by the sensor system, which includes the ISFET microarray chip, microfluidic system, and test platform. The chip achieves a high resolution of at least 0.5 mV, thus enabling the recognition of the change of 0.01 pH value. This complementary metal-oxide semiconductor (CMOS) compatible and cost-efficient sensor array chip, together with other specially designed components, can form a complete DNA sequencing system with potential application in the molecular biology fields.

## 1. Introduction

Deoxyribonucleic acid (DNA) sequencing [1,2], detection of the order of nucleotides in DNA strands, plays a significant role in the field of life and health. DNA sequencing technology provides important data for biomedical research. Gene information obtained by DNA sequencing can be used in clinical medicine diagnosis, personalized medication guidance, disease pathogenesis research, life regulation mechanism research, and other fields [3]. In the mentioned fields, many efforts have been made to develop reliable and cost-effective tools for DNA analysis [4,5]. Meanwhile, the development of faster, cheaper, and more reliable DNA sequencing technology has become the current research hotspot [6,7]. The development of DNA sequencing technology has undergone three generations of sequencing technology, and representative sequencing methods include sanger sequencing [8], pyrosequencing [9], and single molecular sequencing [10]. Most sequencing technologies use optical signals as sequencing signals, although high-throughput sequencing is implemented, the complexity and cost of the required equipment are relatively high.

**Figure 1:**
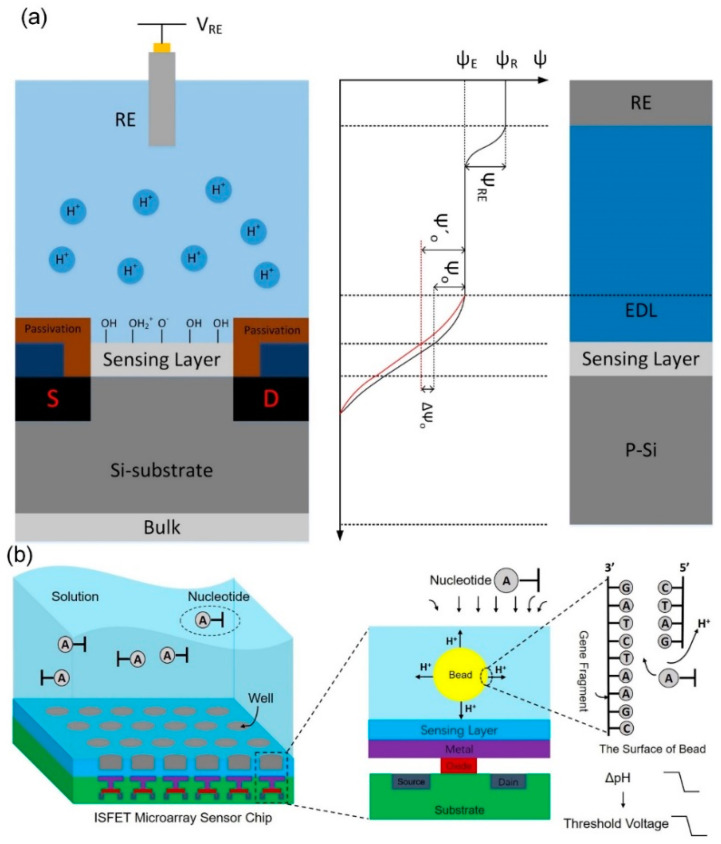
Principles of semiconductor gene sequencing. (**a**) The pH-ISFET structure and working principle, when the pH value in the solution changes, the threshold voltage of the ISFET also changes. (**b**) Sequencing of steps: The long gene chain is cut into small single fragments, which are duplicated, amplified and attached to the microbeads; the microbeads are then distributed into the wells on the surface of the ISFET sensor chip; four kind of nucleotides are supplied in preset order, and when a nucleotide is incorporated into a strand of DNA by a polymerase, a hydrogen ion is released as a byproduct; ISFET sensor senses changes in pH, and the gene sequence then could be obtained.

With the rapid development of microelectronics technology [11], biomedicine [12], microfluidics [13,14], and other frontier technologies, interdisciplinary integration has made biosensors and related application research advance by leaps and bounds. Semiconductor biosensors produced by the cross fusion of very large scale integration (VLSI) technology and biotechnology have great application prospects in many fields, including food, environmental protection, life health, and the Internet of Things [15,16,17,18,19,20,21,22,23,24]. One of the breakthrough applications of semiconductor biosensors in precision medicine is semiconductor gene sequencing, which uses electrical signals instead of optical signals as sequencing signals, thus greatly reducing the complexity and cost of sequencing and making the sequencing process faster. Due to the semiconductor industry Moore’s law development and the advantages of integrated circuits, a semiconductor biochip follows Moore’s law and enables millions of DNA strands to be detected simultaneously by a single chip [25,26,27]. The core sensor detection and data processing capability of a semiconductor gene sequencer depends on a complementary metal-oxide semiconductor (CMOS) array chip, whose basic sensing element is ion-sensitive field-effect transistor (ISFET) [28,29,30,31,32,33,34]. The ISFET is a chemical-sensitive device based on metal-oxide-semiconductor field-effect transistor (MOSFET) structure [35,36,37]. Namely, the ISFET is a MOSFET with chemical sensitivity, so the ISFET structure can be simplified as a field-effect transistor with a removed gate electrode, exposing the gate medium to the solution under the test of the detected ions. The ISFET working principle is to use ions to adsorb on the surface of ISFET sensitive membrane, causing changes in the electric potential of the sensitive membrane/solution interface, thus modulating the FET channel conductance that causes changes in the channel current and converting electrochemical signals into electrical signals. The pH-ISFET sensor system consists of a reference electrode and an ISFET device, where the reference electrode provides a constant reference potential for the solution. The structure and working principle of a pH-ISFET sensor system are shown in Figure 1a. In the solution, the reference electrode provides a constant reference potential (*Ψ_RE_*), and a change in the pH value will cause a change in the potential at the interface of the sensitive film (*Ψ*_0_), as illustrated by the black and red solid lines in Figure 1a represents the change in the electric potential of a solution with the pH value, and *Ψ*_0_ is related to the threshold voltage; the threshold voltage of the ISFET changes proportionally with the pH value of the solution where the sensing layer is immersed. As shown in Figure 1b, when the DNA fragment is attached to the ISFET chip surface, hydrogen ions will be released during DNA synthesis, which can cause a change in the pH value. Due to the ISFET’s sensitivity to hydrogen ions and compatibility with CMOS processes, it is possible to integrate large-scale sensor arrays on a single tiny chip to collect signals in a parallel, high-throughput way continuously. Although the sequencing technology based on the ISFET has proven to be feasible, the sequencing system based on the ISFET microarray sensor chip has not been widely used. Thus, it is of great significance to design an ISFET high integration array chip and the sequencing system based on this chip.

In this study, based on the semiconductor manufacturing international corporation (SMIC) 180 nm CMOS manufacturing process, the ISFET microarray sensor chip is used for pH measurement and DNA detection. More than 13 million sensor array units are integrated on a chip with a size of 15 × 15 mm. More importantly, the microarray sensor chip uses silicon nitride as the sensitive film, which is easy to be compatible with CMOS integrated circuit process, using standard process and low cost. Meanwhile, the chip integrates a signal readout circuit, an addressing circuit, and a bias circuit. The chip package is specially designed and combined with a programmable microfluidic system to realize the precise control of the electrolyte solution on the chip surface. Signal acquisition and signal processing printed circuit board (PCB) perform signal acquisition and processing of a microarray sensor chip. The pH measurement and detection of DNA base pairing are realized. The results show that the chip can achieve a resolution of at least 0.5 mV and a pH sensitivity greater than 70 mV/pH and successfully detect the occurrence of DNA base pairing reaction. The proposed high-density, COMS-compliant, low-cost ISFET array chip is of great significance for DNA detection and sequencing applications.

## 2. Sensor Chip and Test System

### 2.1. System Chip Design and Signal Readout Circuit Design

**Figure 2:**
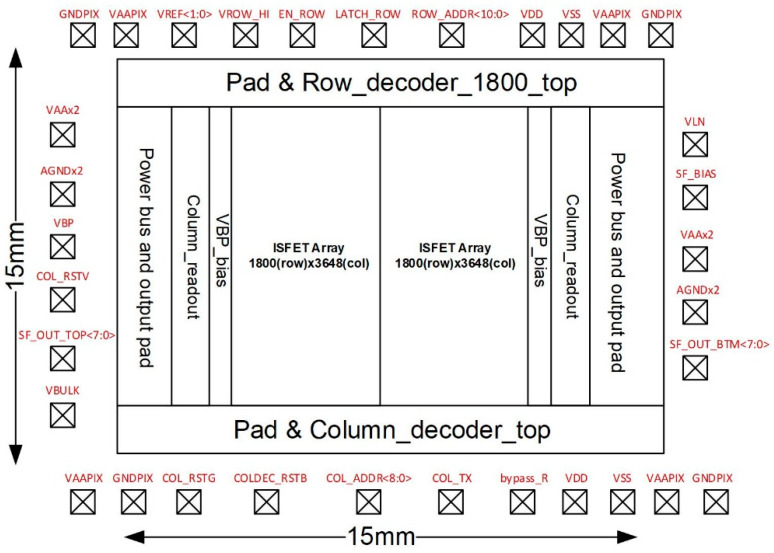
The structure block diagram of the ISFET sensor array chip. The chip can be divided into sensor array unit and read circuit, where the sensor array includes 3600 × 3648 pixel units, and the reading circuit includes row decoder module, column decoder module, VBP bias module, and column readout module.

The sensor chip is fabricated using the standard 180 nm CMOS process. The basic structural block diagram of the chip is presented in Figure 2. The chip has an area of 15 × 15 mm. The sensor array is divided into two parts, each consisting of 1800 × 3648 pixels. More than 13 million sensor array units are integrated into a chip. The modules row decoder and column decoder are used for decoding and addressing the sensor array address. The modules VBP bias and column readout are evenly distributed on both sides of the array. The VBP bias is used to provide a bias voltage to the pixel circuit, and column readout is used to read pixel circuit signals and output them to the sampling circuit outside the chip. The pads of the chip enable the interconnection with external electronics. The chip integrates the signal readout circuit, addressing circuit, and bias circuit.

**Figure 3:**
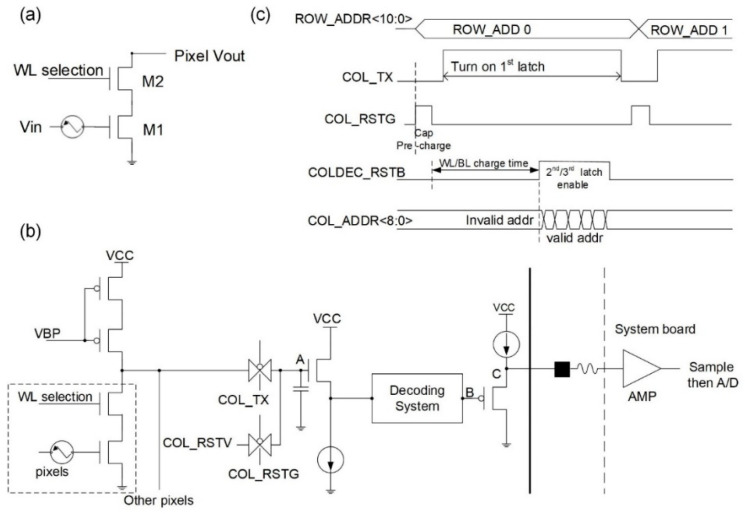
Pixel readout circuit structure and circuit timing diagram. (**a**) Pixel circuit structure; the pixel circuit consists of only two transistors, forming a common-source and common-gate structure. (**b**) Pixel readout circuit structure; when the pH value changes, the output current of the ISFET in the pixel circuit also changes, and the pixel readout circuit converts the current signal into the voltage signal and output it to the external analog-to-digital converter. (**c**) Pixel readout circuit timing diagram; the pixel signals are read according to the timing diagram of the readout circuit; the activation and effective transmission of each pixel unit are controlled by timing.

In order to ensure that the high-density ISFET array can be read quickly, the pixel circuit consists of only two transistors, forming a common-source and common-gate structure, as shown in Figure 3a. In each pixel, M1 denotes an ISFET input tube offset by an external reference electrode, which is a component of a common-source amplifier; M1 tracks changes in the gate potential caused by pH changes and converts them into current signals; M2 is the common-gate tube in the pixel cell circuit, which represents the switch of the pixel cell circuit, controlled by the selection signal (WL selection) generated by the decoder of the system. At the same time, the pixel circuit structure has a certain amplification function, improving the sensitivity of the output voltage to pH, and since no additional amplifying circuit is required, the area of the chip can be smaller.

This study proposes an improved ISFET readout structure that is shown in Figure 3b. The operation sequence of the proposed structure can be explained by the timing diagram, which is illustrated in Figure 3c. When a row of pixels is activated by the WL selection, the nodes A, B, and C will be pre-charged by the transmission gate circuit after COL_RSTG is activated, and the size of the refresh signal will be COL_RSTV. In the pH sensing stage, there will be a discharge path formed by turning on the transmission gate circuit, controlling by COL_TX, and turning off COL_RSTG. As mentioned previously, a change in the pH value will equivalently change the ISFET threshold voltage and the output current, influencing the potential on node A. The signal set up at node A is followed by a source follower device. The source follower device can isolate the effects of node A and other subsequent circuits, thus avoiding the formation of a path between the charge stored at node A and the equivalent capacitance of the following circuit, charge redistribution, and interference with the stored original signal. Then, the decoder circuit determines the construction of the signal at node B; namely, the column decoder determines the column address gate of the signal and controls the sequence output of 3648 columns of the signal built at node A. The signal of node B is sent to node C through another source follower device, which can also isolate the signal of node B from the external signal path; finally, the signal reaches the external signal acquisition circuit through the analog-to-digital converter (ADC). The detailed explanation of some signals is shown in Table 1.

### 2.2. Sensor Chip Packaging

**Figure 4:**
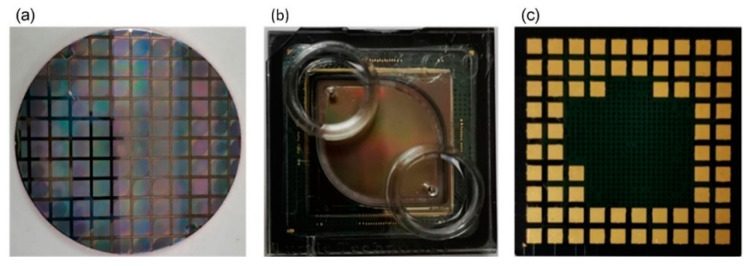
The packaging of the sensor chip. (**a**) The sensor chip is processed on an 8-inch wafer according to the layout design; the wafer is cut into several microarray sensor chips, having the chip area of 15 × 15 mm. (**b**) The front of the sensor chip package, the upper cover, and the chip form a cavity to provide a closed environment; the two holes in the epoxy are the inlet and outlet of the electrolyte solution, which ensure that the solution will not leak during the chip flow. (**c**) The back of the sensor chip package; the chip is put into the chip slot and connected with the external signal acquisition and processing circuit board through the pad.

Since the ISFET microarray sensor chip needs to be in contact with the solution, the chip needs to have a special package structure. Using the SMIC 180 nm CMOS manufacturing processing technology, the sensor chip is processed on an 8-inch wafer, as shown in Figure 4a. The wafer is cut into several microarray sensor chips, and the chip area is 15 × 15 mm. The packaged sensor chip is shown in Figure 4b,c. The chip is wire-bonded to a ceramic package by etching the nitride passivation over the bonding pads by a laser. The function of the epoxy is to isolate the bonding wires and to form a reservoir on the chip surface for solutions. The two holes in the epoxy denote the inlet and outlet of the electrolyte solution, ensuring the solution will not leak during the chip flow. The chip is put into a specially designed chip slot and connected with the acquisition system through the pad on the back of the chip. The chip package is suitable for the specially designed test electronics board.

### 2.3. System Test Platform and Microfluidic Test System

**Figure 5:**
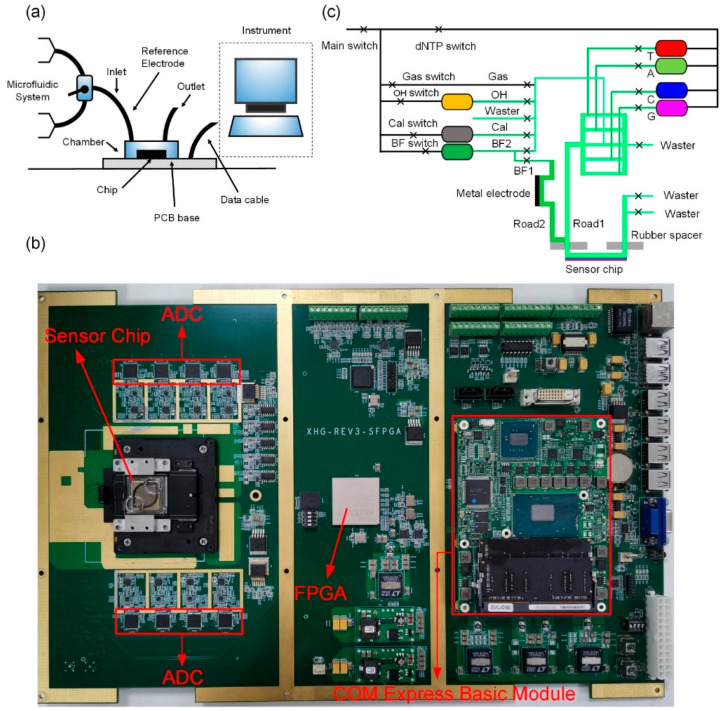
The system test platform and the microfluidic test system. (**a**) The test platform includes the microfluidic system, signal acquisition, and processing system; the signal acquisition and processing system is mainly composed of a high-speed PCB circuit board, which can achieve the acquisition speed of 9.52 ns per pixel, and the reading rate of the array is 128 frames per second. (**b**) Picture of the PCB control board. The main components of the PCB control board are the ADC, FPGA, and COM express basic module. The sensor chip is placed in the chip slot. (**c**) Microfluidic system controls the flow of different solutions by controlling the on and off states of different solenoid valves.

The test platform included the microfluidic system, signal acquisition, and processing system, as shown in Figure 5a. The sensor chip was placed in the chip slot, and the chip was pressed tightly on the PCB control board and interconnected via the pad. The PCB control board mainly included the analog device (AD) data acquisition module, field programable gate array (FPGA) module, and COM express basic module. The picture of the PCB control board is displayed in Figure 5b. The sensor array chip output the analog signal, and the AD acquisition module realized the high-speed acquisition of the analog signal. The FPGA module received the data collected by the AD acquisition module and realized the direct memory access (DMA) function to transfer the collected data to the COM express basic module. The microfluidic system periodically injected solutions into the cavity of the chip through the inlet, and the reference electrode biased the entire sensor array through the contact with the solution. After the chemical reaction occurred, the sensor chip converted the pH change into the voltage change and transmitted it to the COM express basic module through the signal acquisition system, and finally, the processing system processed the data. The system could achieve the acquisition speed of 9.52 ns per pixel, and the readout speed of the array was 128 frames per second. It provided high time resolution, thus accurately monitoring the chemical reaction and pH change.

In order to control the steady flow of the solution on the chip surface, the microfluidic system was a controllable and programable device for conveying fluid in the cavity. The schematic diagram of the microfluidic system is shown in Figure 5c. The rubber spacer was bonded onto the chip using a pressure system, and the chip pad and the pad of the acquisition system were connected together to realize the interconnection of the pins. A metal reference electrode was placed at the entrance to the Road 2 path to provide bias for the entire array chip, as well as for solutions OH and CAL; the solution OH was an alkaline solution and could be used to adjust the pH value of the BF solution; the solution CAL was a standard pH solution, which could be used as a reference to adjust the pH value of the BF solution; the BF solution was a wash solution. Four nucleotides (dNTPs) were cyclically delivered through Road 1 to the chip to cause chemical reactions. All the fluids that flowed through the whole microfluidic system were controlled by different electromagnetic switches whose order could be accurately programable. Liquid solutions could flow through the outlet at different speeds. The physical diagram of microfluidic system is presented in Figure S1.

## 3. Results and Discussion

### 3.1. Electronic Performance

**Figure 6:**
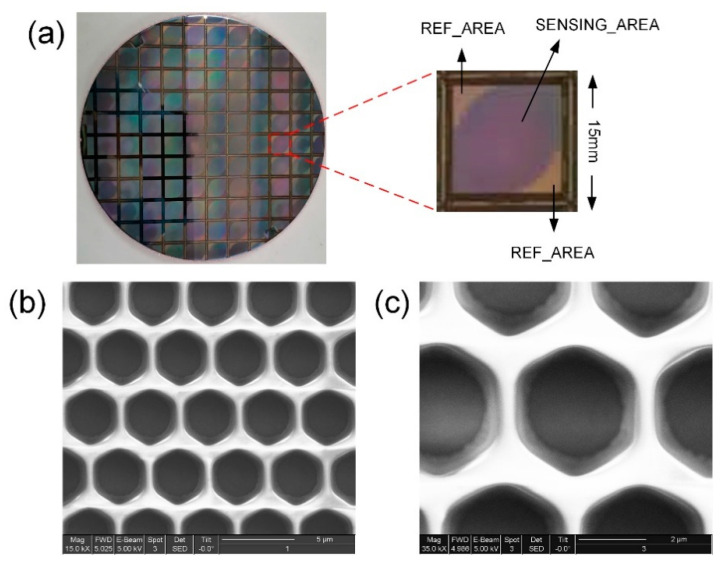
(**a**) The dark bule area in the chip is SENSING_AREA, and the other area is REF_AREA. (**b**,**c**) Scanning electron micrograph of the ISFET microarray.

The electronic performance of the sensor chip was verified before conducting the DNA tests. The most vital performance parameter was the resolution of the chip. The change in the output voltage was measured by applying different step voltages at the reference electrode. The sensor chip was divided into sensing area and reference area; the pixel of the reference area (REF_AREA) was an ordinary MOSFET without sensing function; the pixel of the sensing area (SENSING_AREA) was an ISFET with the pH-sensing function; the pixel of the reference area provided the reference for system measurement. REF_AREA and SENSING_AREA are shown in Figure 6a. The dark blue areas indicate that the upper surface of the chip is covered with silicon nitride. Scanning electron micrographs of the ISFET microarray are shown in Figure 6b,c.

**Figure 7:**
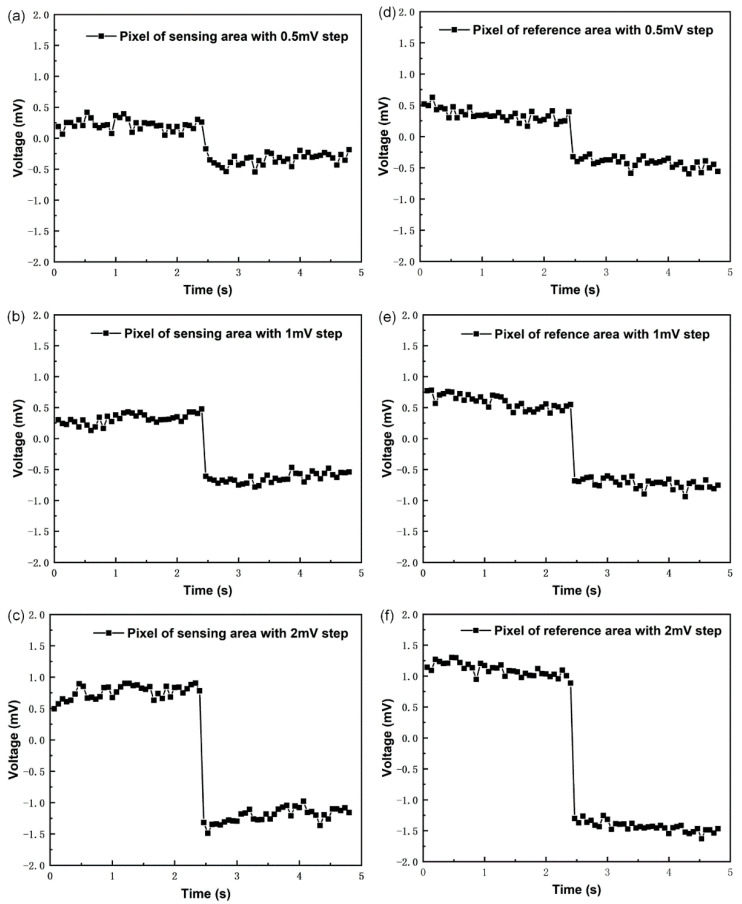
The change in the pixel output voltage with the reference voltage. (**a**) Pixel of the sensing area output at a 0.5 mV step input. (**b**) Pixel of the sensing area output at a 1 mV step input. (**c**) Pixel of the sensing area output at a 2 mV step input. (**d**) Pixel of the reference area output at a 0.5 mV step input. (**e**) Pixel of the reference area output at a 1 mV step input. (**f**) Pixel of the reference area output at a 2 mV step input.

The step voltage of 0.5, 1, and 2 mV was added to the reference electrode. The measured output values in volts are shown in Figure 7. The signal acquisition and processing system collected the output voltage of the chip within five seconds and obtained the change of the output voltage. The sensing area and reference area showed an obvious step output under three different step voltage values. At the step voltage of 0.5, 1, and 2 mV, the output step in the sensing area was 0.65, 1.11, and 2.13 mV, respectively. Furthermore, at the step voltage of 0.5, 1, and 2 mV, the output step in the reference area was 0.8, 1.24, and 2.43 mV, respectively. According to the electronic performance, the proposed chip is suitable for applications requiring resolution less than 0.5 mV. The sensitive film of the ISFET in the sensor chip was silicon nitride, whose sensitivity was about 50 mV/pH, and as the sensing layer, so the sensor chip can recognize the change of 0.01 pH value in the solution. The calculation formula is:

$$\frac{0.5\;\mathrm{mV}}{50\;\mathrm{mV}/\mathrm{pH}}=0.01\;\mathrm{pH}$$

### 3.2. Electrolyte Flow Is Measured on the Sensor Chip Surface

Due to the special packaging mode of the sensor chip, the diffusion rate of the solution on the surface of the chip was uneven, so when the solution diffused in the sensor chip, the output signal time of different sensor units of the chip was different. In this paper, a test method is designed. The surface of the chip was flushed alternately with different electrolyte solutions. The high-speed data acquisition and processing system could quickly collect and store the output signal of the array unit.

**Figure 8:**
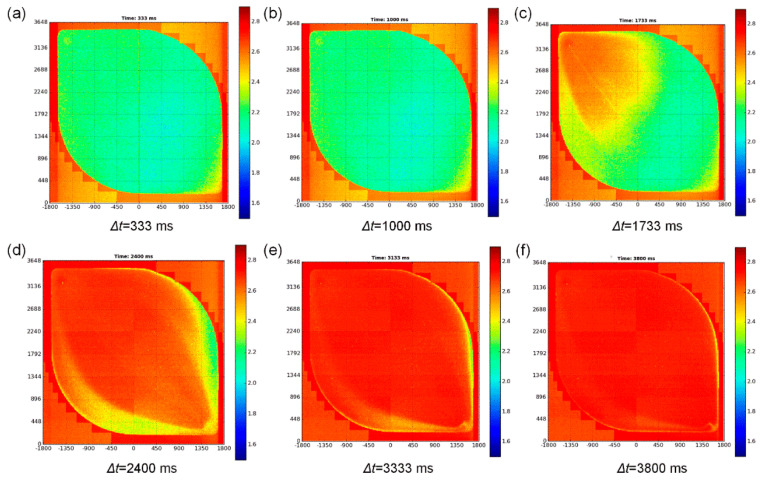
Maps of the sensor chip. The microfluidic system can precisely control the flow of the electrolyte solution on the chip surface; the signal processing and acquisition system can collect every pixel of the sensor array chip in real-time. The output of each pixel of the sensor array chip at a different time: (**a**) Δ*t* = 333 ms. (**b**) Δ*t* = 1000 ms. (**c**) Δ*t* = 1733 ms. (**d**) Δ*t* = 2400 ms. (**e**) Δ*t* = 3333 ms. (**f**) Δ*t* = 3800 ms.

The typical transient response of the sensor array chip is presented in Figure 8, where it can be seen that the data sampling system could record the change in the output signal of the sensor array with time in detail. The data of each pixel were assembled to form complete images as a function of time. The sequence of the output frames is shown in Figure 8, where the imaging resolution is equal to the size of one pixel. The image series in Figure 8 illustrates the flow of the solution above the surface of 3600 × 3648 ISFET sensor pixels in about three seconds. In general, the results showed that the microfluidic system could accurately control the flow of electrolyte solution on the chip surface, but the solution switching took a certain time. The data acquisition and processing system could meet the frequency of chemical reaction, and the sensor chip and signal processing system could meet the requirements of DNA detection.

### 3.3. PH Sensitivity Test and DNA Base Pairing Test

**Figure 9:**
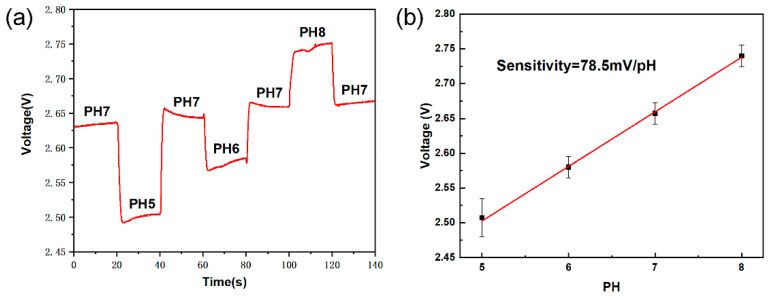
Test the pH sensitivity of the sensor chip. (**a**) The chip has a stable output for different pH values. (**b**) The sensitivity of the chip to the pH value is about 78.5 mV/pH.

In order to test the pH sensitivity of the sensor chip, the buffer solution with a different pH value was continuously injected into the chip surface. As shown in Figure 9a, the sensor chip had different output values for different pH buffers; the duration of each solution was 20 s. The average output of the chip sensor unit is presented in Figure 9a, where it can be seen that the chip had a relatively stable output for different pH values, and the output was also relatively stable after switching with different pH buffers. As shown in Figure 9b, there was a good linear relationship between the chip output and pH, and the slope of the linear relationship was approximately 78.5 mV/pH. High sensitivity of pH can be achieved by circuit amplification, compared with the intrinsic pH sensitivity (50 mV/pH) of ISFET, the pH sensitivity was distinctly improved.

During a sequencing period, four types of nucleotides (dATP, dGTP, dCTP, and dTTP) and a wash solution that flowed into the sensor array were periodically controlled by the microfluidic system. By amplification reaction, millions of strands of DNA are attached to the beads and the beads are distributed into the wells on the surface of the ISFET sensor chip. When the nucleotide was incorporated into the DNA sample, hydrogen ions were released, and the pH of the solution changed. Therefore, it is possible to determine the DNA sequence by checking if an incorporation event has occurred during a flow cycle. At present, the sensing system can realize the detection of DNA base pairing. The detection of base pairing is the most important step in gene sequencing.

**Figure 10:**
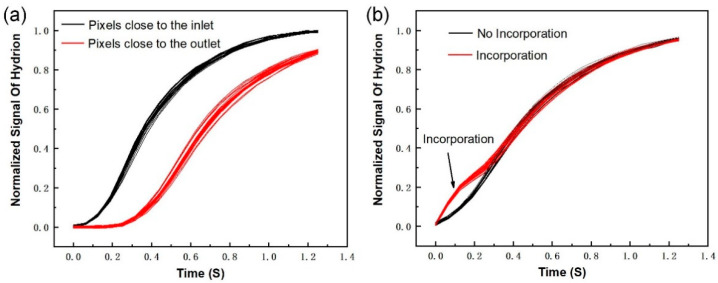
(**a**) When there were no chemical reactions, the pixels only output the background signals. The pixels represented by the black curves were closer to the inlet than the pixels represented by the red curves. (**b**) The distinct red signal bumps that are indicated by the arrow, demonstrate that the chemical reactions to the DNA base pairing have occurred. The DNA base pairing chemical reaction can be detected by the sensor array chip.

During the test with the DNA base pairing, dNTP and washing solution periodically flowed into the surface of the sensor chip to detect whether there were chemical reactions of the DNA base pair incorporation. The curves in Figure 10a represent the output detection result of some pixels. When there were no chemical reactions, the pixels only output the background signals due to reagent change from the wash solution to the dNTP solution. In Figure 10a, the pixels represented by the black curves were closer to the inlet than the pixels represented by the red curves. As shown in Figure 10b, when incorporation events occurred, the pixels output incorporation signals, which had obvious signal bumps at a certain moment relative to the background signals. The arrow in Figure 10b indicates the start of the incorporation events. Thus, it can be concluded that the sensor array chip based on ISFET can be used to detect DNA base pairing.

## 4. Conclusions

In this paper, a 3600 × 3648 ISFET sensor array chip is designed, packaged, and manufactured. A specially designed PCB board and a microfluidic system are implemented. The chip achieves a high resolution of at least 0.5 mV, thus enabling the recognition of the change of 0.01 pH value. The sensing system can realize the detection of DNA base pairing. All the studies are of great significance for the realization of DNA sequencing. Next, we will optimize the sensing system and strive to realize gene sequencing. The microarray gene sequencing technology has gradually become the development trend of sequencing technology. Additionally, the ISFET microarray sensor chip that is compatible with CMOS integrated circuit technology has great potential to be used for gene sequencing.

## Figures and Tables

**Table 1:** Signal name and description.

Signal Name	Description
WL selection	word line selection
VBP bias	constant voltage bias
ROW_ADDR	row address
COL_TX	column enable
COL_RSTG	pre-charge pulse
COL_RSTV	pre-charge voltage
COLDEC_RSTB	column reset
COL_ADDR	column address
AMP	amplifier

## Supplementary Materials

The following are available online at https://www.mdpi.com/article/10.3390/mi12070731/s1, Figure S1: Physical diagram of microfluidic system.
