# Supplementary material for: An ISFET Microarray Sensor System for Detecting the DNA Base Pairing
Source: Micromachines (Basel). 2021 Jun 22;12(7):731. doi: 10.3390/mi12070731 (PMC8305849; doi:10.3390/mi12070731)
Supplement: Supplementary file 1 [file micromachines-12-00731-s001.zip › micromachines-1223590-supplementary.pdf]

micromachines

# Supplementary Information: An ISFET Microarray Sensor System for Detecting the DNA Base Pairing

**Peng Sun [1], Yongxin Cong [1], Ming Xu [1], Huaqing Si [1], Dan Zhao [2] and Dongping Wu [1,*]**

[1] State Key Laboratory of ASIC and System, School of Microelectronics, Fudan University, Shanghai 200433, China; 18112020068@fudan.edu.cn (P.S.); 15210720050@fudan.edu.cn (Y.C.); xuming123@263.net (M.X.); 17112020035@fudan.edu.cn (H.S.)
[2] Shanghai Turtle Technology Company Limited, Shanghai 200439, China; zhaodan@fudan.edu.cn
* Correspondence: dongpingwu@fudan.edu.cn

The reference electrode (RE), such as the widely used Ag/AgCl redox-couple electrode, is both hard to maintain and difficult to integrate as a constituent of an on-chip sensing system. This work demonstrates a microfluidic based on gold or platinum as a metal electrode that can provide a stable reference potential for ISFET-based biochemical sensors. As shown in Fig. S1, the metal electrode is fixed to the microfluidic module. A rubber washer is placed under the metal electrode to ensure that the metal electrode is placed inside the flow system without solution leakage.

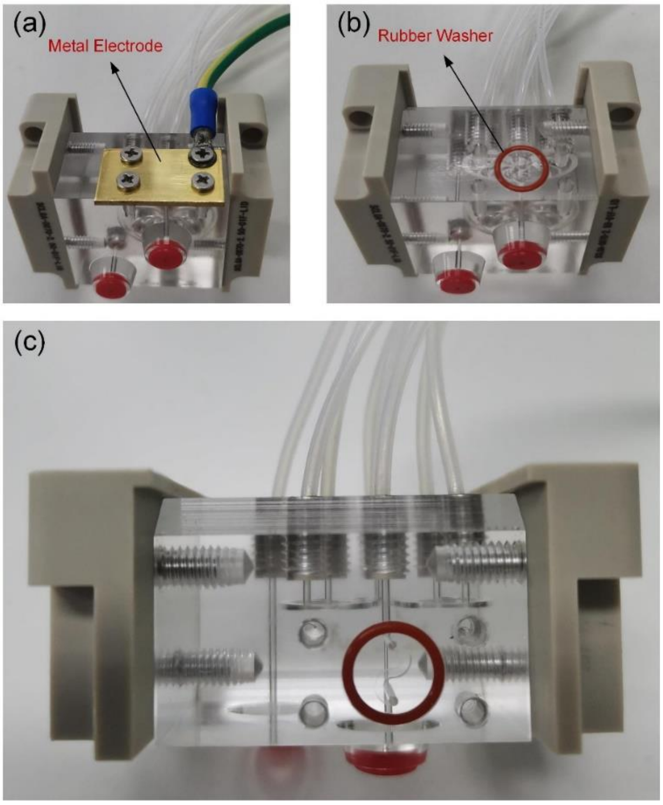


**Figure S1.** Physical diagram of microfluidic system. (a) The metal electrode is fixed to the microfluidic module. (b,c) A rubber washer is placed under the metal electrode to ensure that the metal electrode is placed inside the flow system without solution leakage.
